# LIR and APEAR, two distinct Atg8-binding features within Atg4

**DOI:** 10.18632/oncotarget.17697

**Published:** 2017-05-08

**Authors:** Franziska Kriegenburg, Fulvio Reggiori

**Affiliations:** Fulvio Reggiori: Department of Cell Biology, University of Groningen, University Medical Center Groningen, Groningen, The Netherlands

**Keywords:** autophagy, autophagosome, Atg4, Atg8, LC3

During autophagy, double membrane vesicles called autophagosomes, engulf intracellular structures and deliver them to the vacuole/lysosome for degradation. In addition to its key role in maintaining metabolic homeostasis, autophagy is crucial in eliminating defective or superfluous cellular structures, and consequently impairments in this catabolic pathway cause various diseases. Central components of the machinery mediating autophagosome biogenesis are the members of the Atg8/LC3 protein family, which get conjugated to phosphatidylethanolamine (PE) on the nascent autophagosomal structures [1]. The lipidated form of these proteins drives the elongation and completion of the forming vesicle but also acts as the acceptor for autophagy receptors bound to the cargoes targeted to destruction [1]. Briefly, Atg8 is constitutively processed at its C-terminus by the Atg4 protease to expose a glycine residue, a prerequisite for its conjugation to PE. Atg4 is also essential for Atg8-PE deconjugation from mature autophagosomes, a step promoting the fusion of these carriers with the vacuole/lysosome. As a result, the activity of Atg4 has to be tightly regulated to avoid premature delipidation of Atg8.

**Figure 1:**
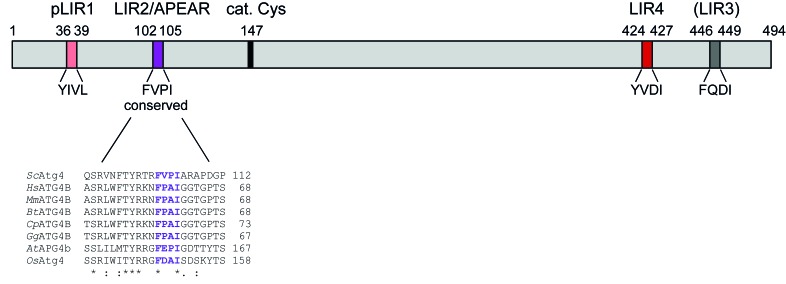
Schematic representation of the distribution of the putative LIR motifs studied in *S. cerevisiae* Atg4 Atg4 consists of 494 amino acids (aa) with the cysteine of the catalytic site (cat. Cys) at aa position 147. Examination of this aa sequence revealed the presence of four putative LIR motifs. Mutations of the different potential LIR motifs showed that pLIR1 (aa 36-39), LIR2/APEAR (aa 102-105) and LIR4 (aa 424-427) are important for the Atg4-Atg8 association *in vivo*. Mutations of the putative LIR3 (gray) at aa position 446-449 had no effect on Atg4 binding to Atg8. While further investigations are still needed to understand the function of pLIR1 (light red), LIR4 (red) was confirmed to be a classical LIR motif as also shown for mammalian ATG4B [6]. The putative LIR2 (violet) at aa position 102-105 proved to be specifically important for Atg4 binding to Atg8-PE but not non-lipidated Atg8. Considering that it does not act like a typical LIR, we named it APEAR for Atg8-PE association region. This region is evolutionary highly conserved as depicted by the fragment aa alignment of *S. cerevisiae* Atg4 (*Sc*Atg4) with ATG4B form various species: *Hs*ATG4B (*Homo sapiens* ATG4B), *Mm*ATG4B (*Mouse musculus* ATG4B), *Bt*ATG4B (*Bos taurus* ATG4B), *Cp*ATG4B (predicted *Crocodylus porous* ATG4B), *Gg*ATG4B (*Gallus gallus* ATG4B), *AtAPG4b (Arapidopsis thaliana ATG4b)* and *OsAtg4 (Oryza sativa Atg4)*.

Our studies in yeast *Saccharomyces cerevisiae* revealed that Atg4 localizes transiently to autophagosomal structures and its recruitment depends on the presence of Atg8 indicating that Atg8 itself engages Atg4 at this site [2]. Since the majority of proteins interact with Atg8 via the so-called LC3-interacting region (LIR) W/F/Y-x-x-L/I/V [3], we scrutinized the yeast Atg4 amino acid sequence and found four potential LIR motifs (Figure 1). Our mutational analysis of these sites proved that three of them are important for Atg4 association with Atg8 *in vivo* [2] (i.e. pLIR1, LIR2/APEAR and LIR4 in Figure 1). However, only one of these putative LIR motifs, the evolutionary conserved site at amino acid position 102-105 (i.e. LIR2/APEAR in Figure 1), is essential for normal progression of autophagy. More precisely, the corresponding Atg4 mutant variant was not efficiently recruited to autophagosomal membranes and displayed a strong impairment in Atg8-PE deconjugation causing a decrease in autophagic flux and also a reduction in autophagosome size [2]. These data confirmed earlier findings showing that the autophagosome size is determined by the amount of available cytosolic Atg8, and directly reflects the ability of Atg4 to delipidate Atg8 [4,5]. Our data about the putative LIR motifs in yeast Atg4 left us with the enigmatic but interesting finding that impairments in Atg4-Atg8 binding do not automatically affect autophagy (i.e. pLIR1 and LIR4 vs LIR2/APEAR in Figure 1). This observation led us to hypothesize that the region mutated in this autophagy-defective Atg4 variant is specifically important to recognize Atg8-PE. Indeed, the conserved region around amino acids 102-105 became dispensable for the Atg4-Atg8 interaction in cells where Atg8 is only present in its non-lipidated form. We confirmed these data *in vitro*, where this mutant Atg4 variant bound to recombinant (non-lipidated) Atg8 as wild type Atg4. We thus identified a distinct region in yeast Atg4, which does not act like a classical LIR motif but rather is particularly important for the association of Atg4 with lipidated Atg8 (Atg8-PE) and hence named it APEAR (Atg8-PE association region) [2]. In our study, we also confirmed that a putative LIR sequence at the C-terminus of Atg4 (i.e. LIR4 in Figure 1) is indeed a LIR motif since it is essential for the *in vivo* and *in vitro* Atg4-Atg8 association [6]. A combinational approach, in which both APEAR and this LIR motif were mutated, revealed a cooperative Atg8-binding mechanism of these two sites.

In summary, our data revealed that different regions within yeast Atg4 are involved in distinguishing between conjugated and non-conjugated Atg8, providing new insight into the regulation of this protease. Very interestingly, the APEAR sequence is conserved among ATG4 proteins (Figure 1, [2]), which also have confirmed LIR motifs on their N- and C-termini (e.g. [6, 9]). Thus, it is imaginable that ATG4 protease function from different species might be regulated by a similar underlying mechanism as the one that we described for yeast Atg4. However, due to the complexity of the LC3/Atg8 and ATG4 system in higher eukaryotes with the presence of several homologous proteins, with different functions in and outside autophagy, other layers of regulation likely exist. So far, it had been shown that redox regulation or C-terminal phosphorylation are involved in the modulation of Atg4 activity [7,8]. Next to this, one could hypothesize that the area around APEAR could also be subjected to modifications such as phosphorylation, e.g. there are highly conserved potential phosphorylation sites present upstream of APEAR (Figure 1). To understand the regulation and mechanism of Atg4 binding to lipidated Atg8 in detail, it will be crucial to determine the structure of Atg4 associated to Atg8-PE as only non-lipidated Atg8/LC3 in complex with Atg4 has been solved so far [9]. One can easily imagine that conjugation to PE results in conformational changes in Atg8, exposing extra binding sequences and/or possibly hiding other ones. This could explain why the APEAR motif is crucial for Atg4 binding to Atg8-PE and why its mutation does not affect the association to non-lipidated Atg8. Future studies are needed to completely decipher Atg4 regulation.
